# Targeting the Interplay Between Autophagy and the Nrf2 Pathway in Parkinson’s Disease with Potential Therapeutic Implications

**DOI:** 10.3390/biom15010149

**Published:** 2025-01-19

**Authors:** Mengru Liu, Siqi Liu, Zihan Lin, Xi Chen, Qian Jiao, Xixun Du, Hong Jiang

**Affiliations:** 1 Department of Physiology, Shandong Provincial Key Laboratory of Pathogenesis and Prevention of Neurological Disorders and State Key Disciplines: Physiology, School of Basic Medicine, Qingdao University, Qingdao 266000, China; mengru-liu@zju.edu.com (M.L.); liusiqi@qdu.edu.cn (S.L.);; 2 Qingdao Key Laboratory of Neurorehabilitation, Qingdao Hospital, University of Health and Rehabilitation Sciences, Qingdao 266113, China

**Keywords:** Parkinson’s disease, autophagy, oxidative stress, Nrf2 pathway, p62

## Abstract

Parkinson’s disease (PD) is a prevalent neurodegenerative disorder marked by the progressive degeneration of midbrain dopaminergic neurons and resultant locomotor dysfunction. Despite over two centuries of recognition as a chronic disease, the exact pathogenesis of PD remains elusive. The onset and progression of PD involve multiple complex pathological processes, with dysfunctional autophagy and elevated oxidative stress serving as critical contributors. Notably, emerging research has underscored the interplay between autophagy and oxidative stress in PD pathogenesis. Given the limited efficacy of therapies targeting either autophagy dysfunction or oxidative stress, it is crucial to elucidate the intricate mechanisms governing their interplay in PD to develop more effective therapeutics. This review overviews the role of autophagy and nuclear factor erythroid 2-related factor 2 (Nrf2), a pivotal transcriptional regulator orchestrating cellular defense mechanisms against oxidative stress, and the complex interplay between these processes. By elucidating the intricate interplay between these key pathological processes in PD, this review will deepen our comprehensive understanding of the multifaceted pathological processes underlying PD and may uncover potential strategies for its prevention and treatment.

## 1. Introduction

Parkinson’s disease (PD), primarily affecting middle-aged and elderly populations, is a highly prominent progressive neurodegenerative disorder. PD patients manifest a wide range of symptoms, such as motor dysfunction, cognitive decline, depression, sleep disorder, and gastrointestinal dysfunction [1]. A hallmark pathological characteristic of PD is the formation of Lewy bodies (LB), which encompass aberrant aggregates of α-synuclein protein, leading to the gradual degeneration of dopaminergic (DAergic) neurons in the substantia nigra (SN). Numerous studies have determined that both environmental factors and genetic mutations contribute to the onset of PD, initiating various pathological processes such as autophagy dysfunction, oxidative stress, proteostasis disruption, mitochondrial dysfunction, and neuroinflammation [2,3]. These processes interact to form a complex network that further drives the progression of PD. Notably, autophagy and oxidative stress are critical pathological processes in PD.

Autophagy is a complex and evolutionarily conserved intracellular process essential for regulating cell proliferation, survival, and death through degrading, recycling, and repurposing cellular components [4,5]. As a fundamental mechanism of cellular degradation, autophagy removes both physiological and pathological α-synuclein, thereby contributing to the maintenance of cellular proteostasis [6,7]. Conversely, in DAergic neurons, deficits in core autophagy genes (e.g., *ATG7*) lead to presynaptic accumulation of α-synuclein, resulting in DAergic neuronal loss and locomotor impairments [8]. Notably, mutations or defects in PD risk genes, as well as exposure to PD-associated neurotoxins, can compromise autophagic functions, which has emerged as a pivotal pathogenic mechanism in PD [9,10].

Oxidative stress, arising from the superfluous reactive oxygen species and insufficient antioxidant defenses, has also been linked to PD pathogenesis [11,12,13]. Oxidative injury to lipids, DNA, RNA, and mitochondria in the brains of PD patients leads to neurodegeneration [14,15,16,17]. Thus, neutralizing oxidative stress could be a promising avenue for PD. Nuclear factor erythroid 2-related factor 2 (Nrf2), a critical transcription factor, is acknowledged as the principal modulator of cellular antioxidant processes, neutralizing various cellular stresses, primarily oxidative stress [18]. In addition, Nrf2 orchestrates several critical cellular processes, including detoxification, proliferation, differentiation, metabolism, and inflammation, enabling cells to effectively counteract various forms of cellular stress [19,20,21]. Notably, in PD models, Nrf2 pathway activation has been shown to alleviate the accumulation of toxic α-synuclein, oxidative stress, and neuroinflammation, thereby reducing DAergic neuronal demise [22,23]. Therefore, Nrf2 pathway has been recognized as a valuable therapeutic target for PD.

Current studies have increasingly highlighted the intricate crosstalk between autophagy and the Nrf2 pathway [24,25,26]. Autophagy modulates Nrf2 through the p62–Keap1 complex, promoting Nrf2 nuclear translocation and subsequent activation of antioxidant gene expression [27,28,29]. Conversely, Nrf2 influences autophagy by regulating the transcription of autophagy-related proteins (p62, ATG5, ATG8), other proteins (Sestrin2, Aldolase C), and miRNAs (e.g., miR-129-3p), as well as additional regulatory molecules [30,31,32,33,34]. Notably, p62, serving as an autophagy adaptor and Nrf2 target protein, can form the p62–Keap1–Nrf2 axis, thereby bridging the interplay between autophagy and Nrf2 crosstalk [28,35]. In neurodegenerative disorders such as PD, the interplay between autophagy and Nrf2 is particularly crucial [36,37,38,39,40]. Autophagy enhances Nrf2 activity, which mitigates oxidative stress, and preserves DAergic neurons. In turn, Nrf2 activation further stimulates autophagy and promotes the removal of α-synuclein aggregates, enhancing neuronal survival in PD [41]. Therefore, synergistic interplay between autophagy and the Nrf2 pathway forms a pivotal defense mechanism in PD. This review describes the involvement of autophagy, the Nrf2 pathway, and their interplay in PD, aiming to provide insights into the interaction of these key pathological processes and offer novel targets for PD therapy.

## 2. Autophagy

Autophagy is a lysosome-driven degradation mechanism that targets cellular proteins and even entire organelles for breakdown and recycling [42]. Autophagy is typically classified into three types based on the mechanism by which cargo is conveyed to the lysosomes: macroautophagy, chaperone-mediated autophagy (CMA), and microautophagy. Macroautophagy, the most thoroughly studied type, is often simply termed autophagy. It involves the generation of double-membraned autophagosomes, which encapsulate misfolded proteins and damaged organelles, subsequently fusing with lysosomes for degradation. In CMA, proteins containing the KFERQ motif selectively interact with molecular chaperones such as heat shock cognate protein 70 (HSC70). Then, the complex is recognized by lysosome-associated membrane protein type 2A (LAMP2A), which is situated on the lysosomal membrane, facilitating the entry of cargo into the lysosome for degradation. In microautophagy, lysosomal membrane directly engulfs cargo by invagination or deformation, followed by degradation within the lysosome [43].

### 2.1. Process of Autophagy

**Figure 1:**
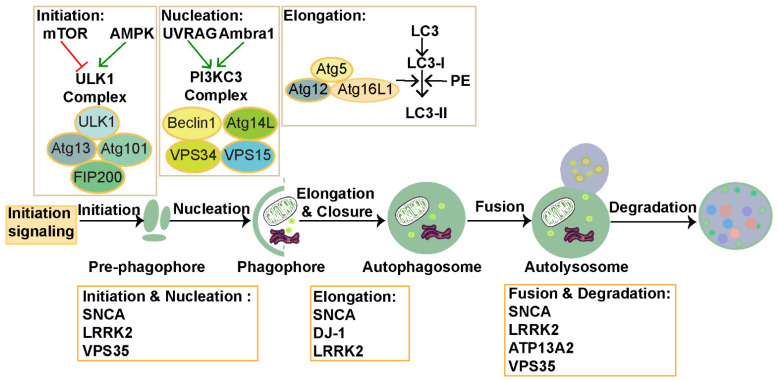
The process of autophagy. The autophagy process is divided into six stages: initiation, nucleation, elongation, closure, fusion, and degradation. (Upper part of the scheme: the green arrows indicate positive regulators, while the red color denotes negative regulators). Various PD risk genes that influence autophagy stages are highlighted in the orange boxes.

Autophagy, a critical cellular self-regulatory process, typically occurs under normal conditions but can be significantly amplified in response to multiple stresses and challenges such as nutrient deprivation, calcium signaling, oxidative stress, and hypoxia [44,45,46,47]. The autophagy process contains six stages: initiation, nucleation, elongation, closure, fusion, and degradation, as illustrated in Figure 1 [4,48]. Approximately 20 core autophagy-related proteins (Atgs), encoded by *ATGs*, orchestrate these stages, ensuring the precise and efficient execution of the autophagy process.

Autophagy initiation is driven by the activation of the Unc-51-like kinase 1 (ULK1) complex, comprising ULK1 (an Atg1 homolog), FIP200 (Atg11), Atg13, and Atg101. The mammalian target of rapamycin complex 1 (mTORC1), consisting of mTOR, Raptor, mLST8, Deptor, and PRAS40, negatively regulates the ULK1 complex [4,49]. Under nutrient-rich conditions, mTORC1 suppresses autophagy by inhibiting ULK1 phosphorylation. By contrast, under nutrient-deprived conditions, mTORC1 becomes inactive, while AMP-activated protein kinase (AMPK) phosphorylates and activates ULK1, thereby initiating autophagy [50].

Subsequent to initiation, the ULK1 complex translocates to the endoplasmic reticulum, facilitating the assembly of the class III phosphatidylinositol 3 kinase (PI3KC3) complex. This complex comprises Beclin1, Atg14L, VPS15, and VPS34. Either Ambra1 or UVRAG interacts with Beclin1, regulating the activation of the PI3KC3 complex [51]. Then, the PI3KC3 complex catalyzes phosphatidylinositol 3-phosphate production on the phagophore membrane, recruiting downstream factors such as the WD-repeat domain phosphoinositide interacting proteins (WIPIs). WIPIs, in turn, recruit the Atg5–Atg12–Atg16L1 complex, which enables Atg8 family proteins (including GABARAP subfamilies and microtubule-associated protein 1 light chain 3 (LC3)) to attach to phosphatidylethanolamine (PE), driving autophagosome elongation [52,53,54].

During the closure stage, the endosomal sorting complex required for transport seals the phagophore, shaping the autophagosome [55]. The fusion of the autophagosome with a lysosome, forming an autolysosome, is mediated by soluble N-ethylmaleimide-sensitive factor attachment protein receptors (SNAREs), homotypic fusion, the vacuole protein sorting complex, and Ras-related proteins (Rabs) [56]. Subsequently, lysosomal hydrolases break down the cargo contained within the autolysosome. The resulting decomposition products are recycled into the cytoplasm for cellular reuse [57].

### 2.2. Fundamental Role of Autophagy

Autophagy is a pivotal mechanism that maintains cellular homeostasis in most eukaryotes. Numerous studies have demonstrated the functions of autophagy. Autophagy can regulate cellular energy metabolism. When exposed to nutrient deprivation, autophagy degrades intracellular components to supply essential nutrients, meet energy requirements, and ensure cell survival [58,59]. Moreover, autophagy continuously removes damaged or superfluous proteins to prevent the accumulation of deleterious proteins, thereby maintaining cellular proteostasis and homeostasis [60,61,62]. Furthermore, autophagy triggers the breakdown of dysfunctional organelles, including mitochondria, peroxisomes, the endoplasmic reticulum, and ribosomes, thereby preventing the accumulation of harmful substances and preserving cellular integrity. For instance, dysfunctional mitochondria and peroxisomes generate excessive reactive oxygen species (ROS), triggering cellular oxidative stress and consequent oxidative injury. Through the activation of selective autophagy, such as mitophagy or pexophagy, cells can selectively eliminate damaged mitochondria and peroxisomes, thereby alleviating ROS production and maintaining cellular health [63]. Intriguingly, current research also shows that some proteins regulating autophagy can orchestrate a range of non-autophagic functions, some associated with membrane functions such as endocytosis, phagocytosis, vesicular trafficking, secretion, and cytokinesis [64,65,66]. Additionally, they can regulate non-membrane functions, including immune responses and inflammation, genomic stability, cell proliferation, and cell death [67,68,69,70]. Consequently, autophagy has been linked to various human diseases, including neurodegenerative disorders, stroke, cancer, and immune disorders. Herein, we focus on the involvement of autophagy in PD.

### 2.3. Dysfunctional Autophagy in PD

It has been widely documented that autophagy is dysfunctional in PD patients and models [71,72,73,74,75]. Genetic analyses have revealed aberrant expression levels of several *ATGs*, including *ATG1*, *ATG5*, *ATG7*, *ATG8*, *ATG16L1*, and *ATG12*, in individuals with PD [76,77,78,79,80,81]. Additionally, in neurotoxin-treated models, such as those exposed to paraquat, 1-methyl-4-phenylpyridinium (MPP^+^), 6-hydroxydopamine (6-OHDA), or 1-methyl-4-phenyl-1,2,3,6-tetrahydropyridine (MPTP), the levels of Atgs are abnormal, indicating compromised autophagy [82,83,84]. The sequestration of proteins LC3 and GABARAPs in LB also signifies a disruption in autophagic flux [85]. Furthermore, altered levels and activities of lysosomal enzymes, such as cathepsin D and glucocerebrosidase (GCase), have been observed in PD patients, suggesting compromised autophagic degradation [86,87,88]. Post-mortem analysis of brain samples from PD patients shows a significant drop in both HSC70 and LAMP2A levels in the SN and amygdala compared to healthy controls, further implying dysfunction in CMA [89].

Dysregulation of autophagy is widely acknowledged as an underlying pathological process in PD. As a crucial mechanism for the degradation of damaged or superfluous proteins, autophagy is essential for the removal of both physiological and pathological α-synuclein [6,90]. Defects in key autophagy core genes can lead to degeneration of DAergic neurons and motor dysfunction. For instance, the absence of autophagy core genes *ATG5* or *ATG7* results in the presynaptic aggregation of proteins such as leucine-rich repeat kinase 2 (LRRK2) and α-synuclein, formation of LB, activation of neuroinflammation driven by the NOD, LRR, and pyrin domain-containing protein 3 (NLRP3) inflammasome, loss of DAergic neurons, and resultant locomotor dysfunction [8,91,92,93]. Conversely, the appropriate activation of autophagy effectively mitigates damage in PD cell and animal models [94]. Activation of autophagy facilitates the removal of damaged mitochondria and α-synuclein aggregates, thereby alleviating oxidative stress and neuroinflammation, thus delaying the pathogenic progression of PD [10,95,96].

### 2.4. PD Genetic Risk Factors Affect Autophagy

A mere 5–10% of PD cases are attributed to genetics, presenting as monogenic forms. Several genes implicated in PD onset, such as *SNCA*, *PARKIN*, *PINK1*, *DJ-1*, *LRRK2*, *ATP13A2*, *VPS35*, and *GBA*, are involved in the modulation of autophagy [97,98,99,100,101,102]. However, further studies on the underlying mechanisms by which PD risk genes regulate autophagy are required. Here, we review how some of the most prevalent PD genes contribute to PD pathogenesis through the regulation of autophagy, as summarized in Figure 1.

#### 2.4.1. *SNCA*

*SNCA*, which encodes α-synuclein, is one of the most extensively studied genes in PD [103]. The pathological accumulation of α-synuclein is a hallmark of PD. Several studies have demonstrated that α-synuclein significantly impairs autophagic steps. For instance, overexpression of α-synuclein enhances the interaction between Bcl-2 and Beclin1, reduces Rab 1a activity, and leads to the mislocalization of Atg9A, thereby compromising the formation of pre-phagophore [104]. Additionally, α-synuclein overexpression reduces the levels of v-SNARE protein SNAP29, which hampers the fusion of autophagosomes with lysosomes [105]. Moreover, α-synuclein overexpression contributes to lysosomal dysfunction, characterized by increased lysosomal pH, reduced lysosome-associated membrane protein type 1 (LAMP1) expression, and altered lysosomal morphology and distribution, all of which impair autophagic degradation [106]. Intriguingly, wild-type α-synuclein modifications such as phosphorylation, ubiquitination, nitrosylation, oxidation, and other post-translational modifications are found in cytosolic aggregates in PD patients and experimental models. These modifications have also been shown to compromise autophagy [107,108]. Taken together, α-synuclein profoundly disrupts key autophagic processes, thereby impairing autophagic flux. This dysfunction leads to the further accumulation of toxic α-synuclein aggregates, which subsequently impair mitochondrial and lysosomal function, trigger neuroinflammation, and synergistically accelerate the progression of PD [109,110,111].

Mutations in the *SNCA* gene, including point mutations such as A53T, A30P, and E46K, and duplications of the entire locus, have been implicated in early-onset familial PD [103,112,113]. Despite variations in clinical presentations, common pathological features such as LB formation, neuronal loss, and disrupted autophagic flux are universally observed [106,114,115,116]. Intriguingly, in the SH-SY5Y cell, PC12 cell, and primary cortical neuron, the mutant α-synucleins A53T and A30P bind to LAMP2A on the lysosomal membrane, thereby suppressing CMA-dependent degradation, leading to the formation of insoluble oligomeric α-synuclein [7,117]. Furthermore, in *SCNA*^A53T^ mice, p38-TFEB disrupts CMA-regulated NLRP3 removal, thereby triggering microglial activation and ultimately leading to DAergic neuronal loss [118]. Moreover, the mutant α-synuclein A53T induces excessive activation of mitophagy, leading to the injury of DAergic neurons [119].

#### 2.4.2. *PARKIN* and *PINK1*

The proteins Parkin RBR E3 ubiquitin-protein ligase (PARKIN) and PTEN-induced putative kinase 1 (PINK1) are encoded by the *PARKIN* (*PARK2*) and *PINK1* (*PARK6*) genes, respectively. It has been documented that PARKIN and PINK1 play pivotal roles in mitochondrial quality control through regulating selective mitochondrial autophagy or mitophagy [120]. Under physiological conditions, PINK1 is processed and rapidly degraded in healthy mitochondria. However, upon mitochondrial damage, PINK1 stabilizes on the outer mitochondrial membrane, where it recruits cytosolic PARKIN. Once recruited, PARKIN ubiquitinates various proteins on the damaged mitochondrial membrane, allowing them to be recognized by autophagy receptors. This orchestrated signaling cascade ensures the selective removal of dysfunctional mitochondria via the autophagy pathway, thereby preserving cellular homeostasis. However, in aged *PARKIN*^−/−^ mice, mitophagy is dysfunctional and further induces stimulation of interferon-gene-mediated neuroinflammation, ultimately leading to DAergic neuronal degeneration [96]. *PARKIN* and *PINK1* mutations hinder the initiation of mitophagy, leading to the accumulation of dysfunctional mitochondria within cells, which contributes to the early onset of autosomal-recessive PD [121,122].

#### 2.4.3. *DJ-1*

*DJ-1* (*PARK7*), encoding DJ-1, was first identified in 1997 and later implicated in early-onset PD in 2004 [123,124]. DJ-1 maintains cellular homeostasis by regulating several essential processes, including mitochondrial and endoplasmic reticulum function, protein homeostasis, and chaperone activity [125,126,127]. Moreover, DJ-1 can regulate autophagy. In microglia, DJ-1 knockdown compromises the autophagy-mediated degradation of LC3, p62, and α-synuclein proteins, as well as increases the levels of pro-inflammatory cytokines interleukin-1β and interleukin-6 [128]. DJ-1 deficiency decreases the number of autophagosome puncta and inhibits the recruitment of LC3 to autophagosomes in SH-SY5Y cells with paraquat treatment [99]. Conversely, DJ-1 overexpression in MN9D cells or rat brains activates the ERK-dependent autophagy pathway, providing neuroprotection against rotenone-induced DAergic neuronal death [129,130]. Additionally, DJ-1 overexpression enhances CMA by upregulating HSC70 and LAMP2A, thereby alleviating the accumulation of α-synuclein [131]. Similarly, astrocyte-specific expression of DJ-1 enhances LAMP2A levels, thereby inducing CMA and alleviating the accumulation and phosphorylation of α-synuclein, oxidative stress, and neuroinflammation, ultimately protecting against rotenone-induced neurodegeneration [132] Furthermore, DJ-1 is pivotal in regulating mitophagy. Wild-type DJ-1 interacts with PINK1, stabilizing it within mitochondria to facilitate mitophagy. In contrast, mutation of DJ-1 promotes PINK1 degradation, leading to compromised mitophagy and subsequent mitochondrial dysfunction, contributing to the pathogenesis of PD [133].

#### 2.4.4. *LRRK2*

LRRK2, encoded by the *LRRK2* (*PARK8*) gene, exhibits kinase activity and regulates synaptic vesicle trafficking and distribution [134]. Mutations in *LRRK2* are the most prevalent genetic contributors to early-onset autosomal-dominant PD [135,136]. Notably, G2019S mutation, the most frequent variant in *LRRK2*, can cause autophagy dysfunction. The G2019S mutation activates leucyl-tRNA synthetase, which subsequently activates the mTORC1 pathway, thereby suppressing the initiation of autophagy [137]. In addition, it has been shown that the G2019S mutation inhibits the co-localization of LC3 with LAMP1, thereby compromising the fusion of autophagosomes with lysosomes [138]. Moreover, the G2019S mutation phosphorylates Rab 10, leading to dysfunctional lysosomes and impairing the autophagic degradation of toxins. Additionally, leaked lysosomal enzyme Cathepsin B co-localizes with the NLRP3 inflammasome, inducing neuroinflammation and subsequent neurotoxicity [139]. A myriad of studies have highlighted that *LRRK2* mutations also lead to the dysfunction of CMA and mitophagy. For instance, in aged *LRRK2*-R1441G knock-in mice, levels of LAMP2A and HSC70, along with the CMA substrate glyceraldehyde 3-phosphate dehydrogenase, are elevated, suggesting that *LRRK2* mutations may interfere with CMA, resulting in inadequate degradation of oligomeric α-synuclein and promoting PD [140]. Moreover, the G2019S or R1441C mutation causes mitochondrial dysfunction and mitophagy defects, contributing to PD pathogenesis [138,141,142].

#### 2.4.5. *ATP13A2*

The *ATP13A2* (*PARK9*) gene encodes an ATPase cation transporting 13A2 (ATP13A2), a lysosomal ATPase that regulates lysosomal homeostasis, mitochondrial and endoplasmic reticulum function, and metal sensitivity [143,144,145,146]. Studies reveal that ATP13A2 can regulate autophagy by recruiting histone deacetylase 6 to lysosomes. Histone deacetylase 6 deacetylates cortactin, thus facilitating the fusion of autophagosomes with lysosomes [147]. Additionally, ATP13A2 regulates PD risk gene *SYT11* in both transcriptional and post-translational manners. Subsequently, SYT11 regulates lysosomal function and enhances the degradation of autophagosomes [101]. In contrast, loss of ATP13A2 leads to dysfunctional lysosomes, p62 aggregation, and neuroinflammation [148]. Additionally, mutations in ATP13A2 impair lysosomal degradation function, primarily due to defects in acidification and reduced proteolytic activity, contributing to the pathogenesis of autosomal-recessive early-onset PD [149].

#### 2.4.6. *VPS35*

Vacuolar protein sorting 35 (VPS35) constitutes an integral component of the retromer complex, which is encoded by the *VPS35* (*PARK17*) gene. VPS35 transports proteins from endosomes back to the Golgi apparatus. *VPS35* mutations are linked to late-onset autosomal-dominant PD [150,151]. The D620N mutation disrupts normal cellular functions by limiting the recruitment of the SCAR homolog complex and Wiskott–Aldrich syndrome protein to endosomes, resulting in abnormal trafficking of the autophagy-related protein Atg9A, which further compromises autophagosome formation [102,152]. Furthermore, the loss or mutation of VPS35 promotes LAMP2A degradation, hampering CMA, and consequently exacerbates the accumulation of α-synuclein in DAergic neurons, thereby contributing to PD pathogenesis [153].

#### 2.4.7. *GBA*

GCase, an essential lysosomal enzyme encoded by the *GBA* gene, accelerates the hydrolysis of glucocerebroside into ceramides and glucose within lysosomes. Mutations in *GBA* are acknowledged as significant and prevalent risk factors for the early onset of PD [154,155]. Loss or mutation of *GBA* results in GCase deficiency, impairing lysosomal function and consequently compromising both autophagy and CMA [154,156,157]. Additionally, in neurons with *GBA* mutations, decreased colocalization between LC3- and LAMP1-positive vacuoles indicates disturbed fusion between autophagosomes and lysosomes [158]. Moreover, heterozygous mutations in *GBA* associated with PD can lead to aberrant mitophagy [159,160]. For instance, the L444P heterozygous mutation reduces levels of the mitophagy adaptor protein BNIP3L/NIX and increases endoplasmic reticulum stress (ER stress), thereby inhibiting mitophagy, contributing to PD pathogenesis [160,161].

## 3. Nrf2 Pathway

Nrf2, known as a primary antioxidant regulator, was identified more than three decades ago. Belonging to the Cap’n’Collar subfamily of basic region–leucine zipper transcription factors, Nrf2 orchestrates the expression of crucial elements within the glutathione–thioredoxin antioxidant systems, alongside enzymes essential for NADPH regeneration, xenobiotic detoxification, and heme metabolism, thereby sustaining cellular redox homeostasis [162,163].

### 3.1. Basic Structure of Nrf2

**Figure 2:**
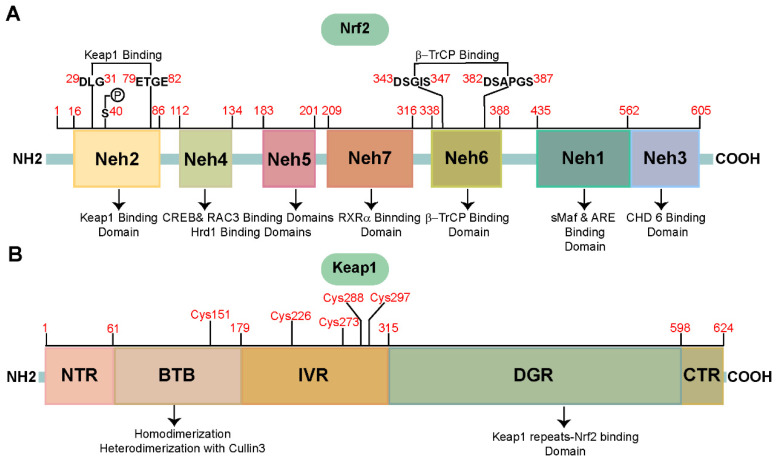
The fundamental structure of Nrf2 and Keap1. (**A**) Seven Neh domains of Nrf2 and their functions. (**B**) Five domains of Keap1, their functions, and several important cysteine residues within different domains that are crucial for Keap1 stability.

Nrf2 is a 68 kDa protein in humans composed of 605 amino acids. Structurally, Nrf2 includes seven distinct functional domains, commonly termed Nrf2–ECH homology (Neh) domains, designated Neh1 to Neh7 [164,165]. The Neh1 domain functions as the binding domain for DNA, where Nrf2 heterodimerizes with small maf proteins (sMaf, MafF, MafG, MafK) and interacts with the antioxidant response element (ARE) on DNA. The Neh2 domain, situated at the N-terminus, is a binding domain for Kelch-like ECH-associated protein 1 (Keap1). Keap1 attaches to the DLG and ETGE motifs of Nrf2, thereby serving as a negative modulator of Nrf2 stability [166]. The Neh3–5 domains function as transactivation domains [167,168,169]. The C-terminal Neh3 domain attaches to the transcription coactivator chromo-ATPase helicase DNA binding protein 6 (CHD6) [167]. Meanwhile, the Neh4 and Neh5 domains can individually or cooperatively engage with cAMP-response element-binding protein (CREB) and repressor-activated coactivator 3 (RAC3) to stimulate Nrf2 target genes transcription [170,171]. The Neh6 domain is recognized by the beta-transducin repeat-containing protein (β-TrCP) via DSGIS and DSAPGS motifs, promoting the ubiquitination and subsequent breakdown of Nrf2 [172]. The Neh7 domain can directly attach to retinoic X receptor alpha (RXRα) to suppress Nrf2–ARE activity [173]. The structure of Nrf2 is illustrated in Figure 2A.

### 3.2. Regulation of Nrf2

#### 3.2.1. Keap1

Keap1, composed of 624 amino acids, functions as an electrophilic sensor and signal transducer, mediating the response to electrophilic compounds and facilitating transcriptional activation. Structurally, Keap1 is delineated into five domains: N-terminal region (NTR); broad-complex, tramtrack, and bric-a-brac (BTB) domain; intervening region (IVR); double glycine repeat (DGR) domain; and C-terminal region (CTR). Among these, the BTB, IVR, and DGR domains are particularly notable as the primary functional domains of Keap1 [174].

In the BTB domain, Keap1 can dimerize, subsequently facilitating its interaction with Nrf2. This domain is also crucial for the assembly of the E3 ubiquitin ligase complex through its interaction with Cullin 3, facilitating the ubiquitination and ensuing breakdown of Nrf2 [175]. Additionally, in the BTB domain, structural modifications of cysteine residues (such as Cys151) can inhibit Nrf2 ubiquitination and degradation, enabling Nrf2 to dissociate from Keap1 and initiate the transcription of ARE genes [176,177].

The IVR region, enriched with cysteine residues, is crucial for maintaining Nrf2 stability. Under basal conditions, this region actively mediates the breakdown of Nrf2. Nevertheless, when exposed to oxidative stress, the cysteine residues of Keap1 (Cys226, Cys273, Cys288, and Cys297) undergo oxidation, promoting Nrf2 release from Keap1. Subsequently, free Nrf2 migrates to the nucleus and initiates ARE gene transcription [178,179,180].

The DGR domain, commonly termed the Kelch domain, comprises six Kelch repeats and serves as the Keap1 binding domain for Nrf2. Under normal conditions, the Kelch domain interacts with Nrf2 via the ETGE motif (higher affinity) and the DLG motif (lower affinity). This interaction facilitates Keap1–Nrf2 complex formation, which is continuously degraded via the ubiquitin–proteasome system (UPS), thereby negatively regulating Nrf2 activity [181]. The basic Keap1 structure is illustrated in Figure 2B.

#### 3.2.2. p62

**Figure 3:**
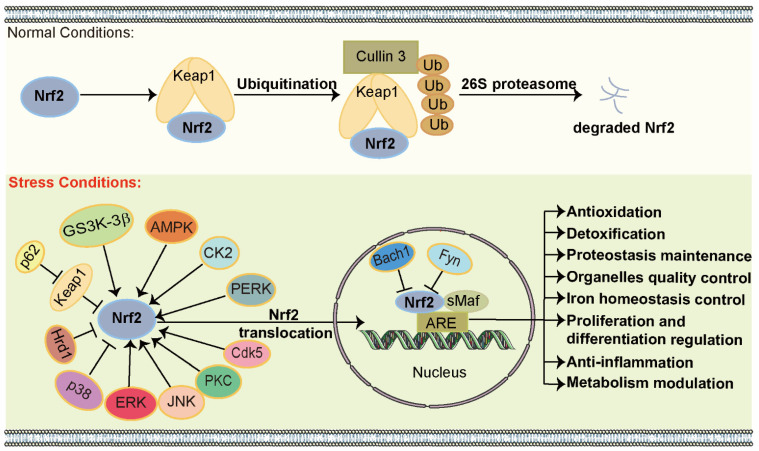
The regulation and fundamental role of Nrf2. Under normal conditions, Keap1 attaches to Nrf2, sequestering it in the cytoplasm and facilitating its degradation through the UPS. However, under stress, various regulatory factors modulate Nrf2, leading to its translocation into the nucleus, where it triggers the transcription of ARE-related genes and thereby regulates numerous cellular functions.

p62 is an adaptor protein that recruits intracellular substrates for degradation via autophagy or UPS [182]. It has been established that p62 regulates Nrf2 activity by recruiting and sequestering Keap1 through its Keap1-interacting region (KIR), which contains the STGE sequence similar to the ETGE sequence of Nrf2. This interaction enables Nrf2 to be released from Keap1, promotes Nrf2 migration to the nucleus, and initiates ARE gene expression [31,183,184]. Upon disruption of autophagy, p62 undergoes impaired degradation, leading to its intracellular accumulation [185,186,187]. Under normal conditions, Keap1 attaches to Nrf2, effectively sequestering it in the cytoplasm and preventing its translocation to the nucleus, positioning Keap1 as a key negative regulator of Nrf2. However, accumulated p62 competes with Nrf2 for binding to Keap1, forming a p62–Keap1 complex. This competition frees Nrf2, allowing it to translocate into the nucleus and initiate ARE-mediated transcription (Figure 3) [188,189]. Intriguingly, recent studies reveal that the activation of autophagy promotes the expression of Nrf2 target genes by accelerating the degradation of the p62–Keap1 complex in neurons. Nrf2 activation, in turn, elevates p62 levels in the cytoplasm, thereby forming a positive feedback loop that defends against oxidative stress [27,36,190]. Therefore, activation of the p62–Keap1–Nrf2 axis provides protective effects against various diseases.

Notably, post-translational modifications of p62 are required for Nrf2 stability. Phosphorylation and deubiquitination of p62 can stabilize p62 and enhance Nrf2 activation. Several kinases can phosphorylate p62, including fructokinase A, mTORC1, protein kinase C (PKC), and ULK1 [25,191,192,193]. In Huh7 and Hep3B cells, fructokinase A phosphorylates p62 at the Ser28 residue, which blocks the ubiquitination of p62, leading to increased binding between p62 and Keap1, and thus activates Nrf2. In MCF-7 cells, PKC phosphorylates p62 at Ser349, facilitating the degradation of the p62–Keap1 complex and resulting in Nrf2 activation. Both mTORC1 and ULK1 also phosphorylate p62 at Ser349, further promoting Nrf2 activation. Furthermore, ubiquitin-specific protease 13 increases p62 stability through the removal of K48- and K63-linked ubiquitin chains from p62 at residue K7, thereby enhancing the autophagic removal of the p62–Keap1 complex and promoting the stabilization and activation of Nrf2.

#### 3.2.3. Glycogen Synthase Kinase-3 Beta (GSK-3β)

GSK-3β, widely expressed in the central nervous system, is a serine–threonine protein kinase that maintains cellular homeostasis by regulating glycogen synthesis, cell proliferation, and cell death [194]. Its enzymatic activity is tightly controlled by inhibitory phosphorylation at the Ser9 residue [195]. Extensive evidence underscores that GSK-3β can modulate Nrf2 activity (Figure 3). Specifically, activation of the PI3K–Akt pathway can phosphorylate GSK-3β at Ser9, leading to the suppression of GSK-3β and an indirect activation of Nrf2. Moreover, the inactivation of GSK-3β has been shown to enhance Nrf2 stability and upregulate the transcription of Nrf2 target genes [196,197]. AMPK can also phosphorylate GSK-3β at Ser9, thereby enhancing Nrf2 activation [198]. Additionally, AMPK can directly phosphorylate Nrf2 at Ser558, located within the nuclear export signal, promoting Nrf2 nuclear accumulation [199]. However, GSK-3β phosphorylates the Src-family kinase Fyn at specific threonine residues, promoting its nuclear localization. Once in the nucleus, Fyn can phosphorylate Nrf2 at Tyr568, resulting in increased nuclear export and subsequent degradation of Nrf2 [200,201]. Additionally, GSK-3β phosphorylates Nrf2 at the DSGIS residues (334–338) of the Neh6 domain, enhancing its ubiquitination by β-TrCP/Cullin 1, subsequently leading to the proteasomal degradation of Nrf2 [202].

#### 3.2.4. HMG-CoA Reductase Degradation Protein 1 (Hrd1) and BTB Domain and CNC Homolog 1 (Bach1)

Hrd1, also known as synoviolin, is an E3 ubiquitin ligase embedded in the endoplasmic reticulum membrane. It maintains protein stability by ubiquitinating misfolded proteins for degradation under ER stress and oxidative stress. Notably, Hrd1 is markedly upregulated in patients with liver cirrhosis and in mice with kidney ischemia–reperfusion injury. Furthermore, Hrd1 attaches to Nrf2 via the Neh4 and Neh5 regions, facilitating its ubiquitination and consequently leading to Nrf2 degradation (Figure 3). Hence, Hrd1, in a Keap1-independent manner, negatively modulates Nrf2 activity, thereby exacerbating liver cirrhosis and kidney ischemia–reperfusion injury [203,204]. Given that Nrf2 primarily functions as a nuclear protein, the mechanism by which it interacts with Hrd1 remains enigmatic, particularly since Hrd1 is chiefly recognized for its role in the degradation of ER-associated proteins. One potential explanation for this interaction lies in the two FxxxFxxxF motifs (F represents phenylalanine, x represents any amino acid) within the Neh7 domain of Nrf2, analogous to motifs found in G-protein-coupled receptors that bind to ER-associated proteins. Alternatively, the interaction with Nrf2 may be mediated by the proline-rich C-terminal tail of Hrd1, which faces the cytoplasm, potentially binding to Nrf2 and facilitating its ubiquitination [205,206]. The expression of Hrd1 is also elevated in 6-OHDA-induced PD models [207,208]. However, in contrast to the cellular damage observed in peripheral diseases, overexpression of Hrd1 alleviates ER stress and thus mitigates 6-OHDA-induced neuronal cell death [208]. Whether and how Hrd1 regulates Nrf2 in PD and other central-nervous-system-related diseases remain to be determined.

Bach1, widely expressed in mammalian tissues, belongs to the Cap’n’Collar subfamily of basic region–leucine zipper transcription factors. Bach1 can competitively bind to sMaf, thereby suppressing Nrf2–ARE transcription (Figure 3) [209,210]. Moreover, a functional ARE region is located close to the initiation site of transcription for Bach1 transcript variant 2 [211]. Therefore, both Nrf2 overexpression and Nrf2-activating agents have been shown to upregulate Bach1 expression. This suggests that Bach1 functions as a regulatory inhibitor of Nrf2 under normal conditions [212]. However, under oxidative stress, which induces Bach1 nuclear export and degradation, Nrf2 activity subsequently restores Bach1 levels, highlighting a feedback mechanism between these two factors.

#### 3.2.5. Phosphorylation of Nrf2

Nrf2 activity is controlled by various mechanisms, with kinase-mediated phosphorylation playing a pivotal role in its post-translational regulation (Figure 3). Several kinases can regulate Nrf2 phosphorylation, including AMPK, casein kinase 2 (CK2), protein kinase R-like endoplasmic reticulum kinase (PERK), cyclin-dependent kinase 5 (Cdk5), PKC and mitogen-activated protein kinases (JNK, p38, and ERK). AMPK phosphorylates Nrf2 at Ser558 residue within the Neh1 domain, promoting Nrf2 nuclear accumulation [199,213]. CK2 has been documented to phosphorylate Nrf2 at several serine and threonine residues within its Neh4 and Neh5 domains, thereby inducing Nrf2 translocation to the nucleus and activating ARE transcription [214]. PERK-triggered phosphorylation of Nrf2 facilitates the disassembly of the Nrf2–Keap1 complex and inhibits their subsequent reassociation. Although the specific phosphorylation sites targeted by PERK on Nrf2 have not yet been identified, it is speculated that these sites reside within the Neh2 domain, leading to disruption of the Nrf2–Keap1 interaction [215]. Cdk5 phosphorylates Nrf2 at the Thr395, Ser433, and Thr439 residues, which enhances Nrf2 nuclear localization and induces ARE transcription [216]. PKC phosphorylates Nrf2 at Ser40, triggering the disassembly of the Nrf2–Keap1 complex, thereby enhancing Nrf2’s antioxidant capacity [217]. Similarly, JNK and ERK can phosphorylate Nrf2 at N-terminal serine residues, triggering Nrf2 pathway [218]. However, Nrf2 phosphorylation by p38 may negatively modulate Nrf2 activity by inducing the NF-κB pathway [219].

### 3.3. Fundamental Role of Nrf2 Pathway

The Nrf2 pathway is a crucial mechanism that sustains cellular homeostasis, as shown in Figure 3. Numerous studies have elucidated the antioxidant functions of the Nrf2 pathway through the modulation of various antioxidant enzymes’ expression. When cells are exposed to oxidative stress, Nrf2 activates the transcription of ARE genes encoding antioxidant enzymes such as catalase, glutathione peroxidase, and superoxide dismutase. These enzymes expeditiously mitigate ROS levels through their catalytic capacities, thereby protecting cells from oxidative injury [220]. Moreover, the Nrf2 pathway is involved in cellular detoxification processes by enhancing the levels of phase II detoxifying enzymes like glutathione S-transferase and NAD(P)H oxidoreductase 1 (NQO1), which metabolize and eliminate harmful exogenous and endogenous substances, thus preventing cellular damage and maintaining cellular homeostasis [221]. In addition, Nrf2 maintains proteostasis by regulating the transcription of molecular chaperones such as heat shock protein 70 and proteasome subunits like 20S proteasomal subunits. These components assist in the proper folding, repair, and degradation of proteins, including α-synuclein and amyloid beta-protein, thereby preventing the aggregation of misfolded or damaged proteins [40,222]. Consequently, Nrf2 is vital for preventing neurodegenerative diseases, for example, Alzheimer’s disease and PD. The Nrf2 pathway also governs the quality control of organelles such as mitochondria, the endoplasmic reticulum, lysosomes, and peroxisomes by regulating selective autophagy or organelle biogenesis [25,223,224,225,226]. Studies have shown that the Nrf2 pathway mediates the transcription of ferritin, thereby controlling iron homeostasis [227]. Nrf2 orchestrates cellular proliferation and differentiation by upregulating the expression of pivotal factors, such as Notch1 and c-Myc [228,229,230]. Furthermore, Nrf2 regulates cellular inflammation, primarily through the attenuation of pro-inflammatory cytokine transcription, including interleukin-6 and tumor necrosis factor-α, as well as the suppression of inflammation-associated enzymes, such as cyclooxygenase-2 and inducible nitric oxide synthase. Additionally, Nrf2 attenuates inflammatory responses by upregulating the expression of IκB, a key negative regulator of NF-κB, and by reducing ROS levels, thereby effectively inhibiting the NF-κB signaling cascade [20,231,232]. Furthermore, Nrf2 regulates cellular metabolism by controlling the transcription of metabolic enzymes. For instance, Nrf2 modulates the pentose phosphate pathway by promoting the transcription of p53-induced glycolysis and apoptosis regulator, transketolase, and glucose-6-phosphate dehydrogenase [233,234]. Additionally, Nrf2 regulates lipid metabolism by influencing genes related to fatty acid synthesis and oxidation, such as phospholipase A2, acetyl-CoA thioesterases, and carnitine palmitoyltransferase 1A [235]. Taken together, the multifaceted role of Nrf2 in cellular defense and homeostasis underscores its potential therapeutic implications for several human diseases.

### 3.4. Involvement of the Nrf2 Pathway in PD

Canonically, Nrf2 resides in the cytoplasm of DAergic neurons in the SN. However, a significantly increased nuclear Nrf2 level has been found in the brains of post-mortem PD individuals [236]. Meta-analyses indicate that approximately 54 Nrf2–ARE-driven genes are altered in PD patients, with 31 genes significantly decreased. Although several Nrf2 target genes, such as p62 and NQO1, are upregulated, they are partially sequestered in LB [237,238]. These studies clarify that the Nrf2 pathway is abnormal in PD.

Numerous PD risk genes impair the effective activation of the Nrf2 pathway, thereby compromising its neuroprotective capacity. For instance, robust immunofluorescence of nuclear Nrf2 in neurons, accompanied by aberrant antioxidant and inflammatory gene responses, is observed in the *SNCA*^A53T^ mouse model [239]. The *SNCA* mutation also prevents the binding of α-synuclein to PKC, resulting in impaired activation of the Nrf2 pathway and contributing to PD pathogenesis [240]. The PINK1 G309D mutation reduces the phosphorylation of Akt and p38, inhibiting Nrf2 translocation and decreasing heme oxygenase 1 (HO-1) levels [241]. DJ-1 knockdown fails to trigger the PI3K–Akt–GSK-3β pathway, leading to an inactive Nrf2 pathway, which contributes to neuronal injury [242,243]. The LRRK2 G2019S mutation increases Keap1 expression and reduces Nrf2 activity, exacerbating oxidative stress and DAergic neuron injury [244,245]. In contrast, microglia-specific loss of LRRK2 upregulates the Nrf2 pathway, thereby alleviating oxidative stress and neuroinflammation [246]. The inhibition of GCase hampers the Nrf2-driven detoxification pathway in neurons [247]. Nevertheless, current research exhibits an inadequate understanding of the precise mechanisms through which various PD risk genes impact the Nrf2 pathway. Moreover, although alterations in the Nrf2 pathway have been documented in PD models, effective strategies to comprehensively restore or augment Nrf2 pathway function to neutralize DAergic neuronal injury remain lacking.

Numerous studies have underscored the pivotal role of the Nrf2 pathway in PD [22,248,249,250]. Genetic variations in the *NFE2L2* gene directly contribute to idiopathic PD [237,238]. Notably, single nucleotide polymorphisms of the *NFE2L2* gene can delay the onset of PD. The expression of Nrf2 and its target genes, such as glutathione reductase, NQO1, and glutamate–cysteine ligase, are elevated in PD patients [251]. However, these increases fail to mitigate excessive oxidative stress. Nrf2 knockout DAergic neurons are more susceptible to MPTP or 6-OHDA-induced neurotoxicity [252,253]. Similarly, Nrf2 knockout exacerbates oxidative stress, the phosphorylation and oligomerization of α-synuclein, and inflammation, ultimately leading to dopaminergic neuron injury in the brains of mice overexpressing human α-synuclein [254]. Conversely, overexpression of Nrf2 confers neuroprotection in SH-SY5Y cells with paraquat treatment [255]. Intriguingly, specific overexpression of Nrf2 in astrocytes or microglia can prevent DAergic neuronal damage in mice treated with neurotoxins, implying that activation of Nrf2 pathway in astrocytes or microglia also contributes to the survival of DAergic neurons in PD [252,256,257,258]. Additionally, numerous regulators of Nrf2 are involved in PD. For instance, deletion of Keap1 constitutively activates the Nrf2 pathway, which alleviates glutamate or rotenone-induced oxidative stress [259]. Likewise, activation of the p62–Keap1–Nrf2 axis attenuates DAergic neuronal death in PD models [27,36]. Furthermore, Nrf2 activators can mitigate PD-related injury. For instance, dimethyl fumarate (DMF), an oral therapeutic drug for multiple sclerosis treatment, functions as an Nrf2 activator by interacting with Keap1. DMF alleviates oxidative stress and immune responses, enhances mitophagy, and thus exerts neuroprotection in SH-SY5Y cells and mouse models of PD [41,260,261]. Therefore, activation of the Nrf2 pathway could be a promising avenue for PD therapy.

## 4. The Interplay Between Autophagy and the Nrf2 Pathway

A number of investigations have elucidated the intricate crosstalk between autophagy and the Nrf2 pathway. As previously mentioned, autophagy regulates the Nrf2 pathway primarily through the p62–Keap1 axis. It has been documented that liver-specific depletion of key autophagy proteins, such as Atg5 and Beclin1, disrupts autophagic function, leading to increased binding of p62 to Keap1 in the cytoplasm, which, in turn, promotes prolonged activation of Nrf2 [29,262]. ATG7 deficiency elevates the levels of p62, alongside the activation of Nrf2 [185,263]. Overexpression of p62 promotes the generation of the p62–Keap1 complex, which significantly activates the Nrf2 pathway [28,264]. However, several studies suggest that autophagy activation can also induce the Nrf2 pathway. Studies have clarified that when cells are exposed to ROS, phosphorylation of p62 in its KIR domain significantly enhances its binding affinity with the DGR domain of Keap1 [25,27,265]. Subsequently, p62 binds to LC3 through the LC3 interaction region, forming the LC3–p62–Keap1 complex, which transports Keap1 to autophagosomes for degradation [266,267,268]. This binding reduces the formation of the Keap1–Cullin3 complex, thereby inhibiting its removal through the UPS, ultimately leading to the activation of the Nrf2 pathway. Thus, autophagy regulates the Nrf2 pathway by controlling the half-life of Keap1 via p62 [25,27,268,269].

Furthermore, other forms of autophagy, including CMA and mitophagy, play an integral role in the activation of the Nrf2 pathway. CMA directly targets and degrades Keap1, thereby initiating Nrf2 activation [270]. Additionally, BNIP3-mediated mitophagy facilitates the clearance of the LC3–p62–Keap1 complex, thus promoting Nrf2 activation [271]. Moreover, prohibitin 2 (PHB2), an essential protein of the inner mitochondrial membrane involved in mitophagy, regulates the expression of Nrf2 and its downstream targets, including HO-1 and NQO1 [272].

In turn, the Nrf2 pathway can regulate autophagy. The Nrf2 pathway directly modulates autophagy by controlling the transcription of ATGs, including *ATG5*, *ATG8*, and *p62*, which possess ARE sequences in their promoters. This regulation upregulates the expression of Atg5, LC3, and p62 proteins [30,31]. Furthermore, Nrf2 orchestrates its target genes to regulate autophagy. For instance, Sestrin2 activates AMPK and suppresses mTORC1, thereby inducing autophagy [32,273,274]. Tripartite motif-containing protein 16 (TRIM16) binds to ULK1 and Beclin1 to trigger autophagy [275,276]. Nrf2 also regulates key molecules, such as aldolase C, to trigger autophagy [33]. Additionally, Nrf2 can regulate microRNAs to modulate autophagy. For example, Nrf2 enhances the level of miR-129-3p, which suppresses mTOR and initiates autophagy [34]. Moreover, Nrf2 negatively regulates miR-214-3p level, whereas miR-214-3p compromises autophagy by targeting *ATG3* [277,278]. Nrf2 upregulates miR-29 (miR-29a and miR-29b) levels, while miR-29 targeting *ATG9A* inhibits autophagy [279,280]. These findings suggest that the Nrf2 may orchestrate autophagic processes at the miRNA level, although direct evidence to substantiate this regulatory mechanism remains largely unknown.

Notably, Nrf2 also orchestrates other types of autophagy, including CMA and mitophagy. For instance, Nrf2 transcriptionally regulates the expression of LAMP2A, thereby activating CMA [223,270]. Additionally, Nrf2 regulates the transcription and expression of PINK1 by binding to AREs located within the upstream promoter of the *PINK1* gene [281]. Moreover, Nrf2 activates PINK1/PARKIN-mediated mitophagy [282]. Furthermore, Nrf2 binds to and regulates the mitophagy receptor optineurin, thereby facilitating the clearance of dysfunctional mitochondria [283]. Nrf2 also regulates the expression of PHB2, thereby further inducing mitophagy [284]. Additionally, Nrf2 enhances BNIP3-mediated mitophagy, although the underlying mechanism requires further investigation [41,285].

**Figure 4:**
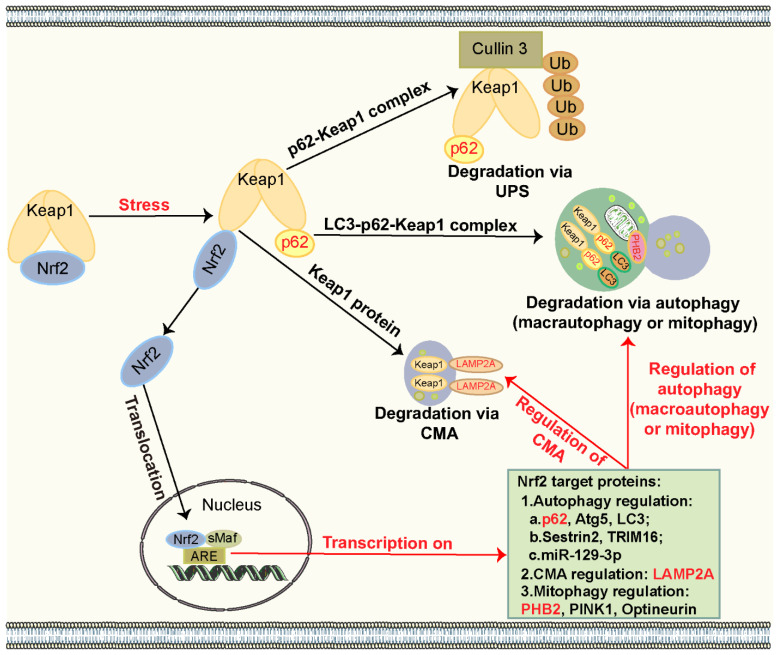
The interplay between autophagy and the Nrf2 pathway. Under normal conditions, Nrf2 interacts with Keap1 and is continuously degraded in the cytoplasm. Upon exposure to cellular stress, Keap1 preferentially binds to p62, leading to the formation of the p62–Keap1 complex. This complex undergoes degradation via the UPS or autophagy, including macroautophagy and mitophagy. Additionally, Keap1 can be directly degraded through CMA. Consequently, Nrf2 is released and translocates into the nucleus, where it dimerizes with sMaf to initiate the transcription of ARE genes. The activation of the Nrf2 pathway can regulate its target genes, which, in turn, modulate various forms of autophagy, including macroautophagy, mitophagy, and CMA.

Taken together, the regulation between autophagy and the Nrf2 pathway is reciprocal. Intriguingly, p62, both an autophagic receptor and a target protein of Nrf2, acts as a bridging factor, establishing a positive feedback loop via the p62–Keap1–Nrf2 axis, thereby mediating autophagy and Nrf2 pathway crosstalk, while PHB2-mediated crosstalk between mitophagy and the Nrf2 pathway is similarly dependent on the p62–Keap1–Nrf2 axis. Likewise, LAMP2A establishes a positive feedback loop through the LAMP2A–Keap1–Nrf2 axis, thereby regulating the interplay between CMA and the Nrf2 pathway. These intricate feedback mechanisms underscore the importance of such interaction in maintaining cellular homeostasis during stress [35,266,267,270,272,286]. However, the precise mechanisms underlying this interplay remain to be clarified. The autophagy and Nrf2 pathway interplay is illustrated in Figure 4.

## 5. Mechanisms and Therapeutic Potential of the Interplay Between Autophagy and the Nrf2 Pathway in PD

**Figure 5:**
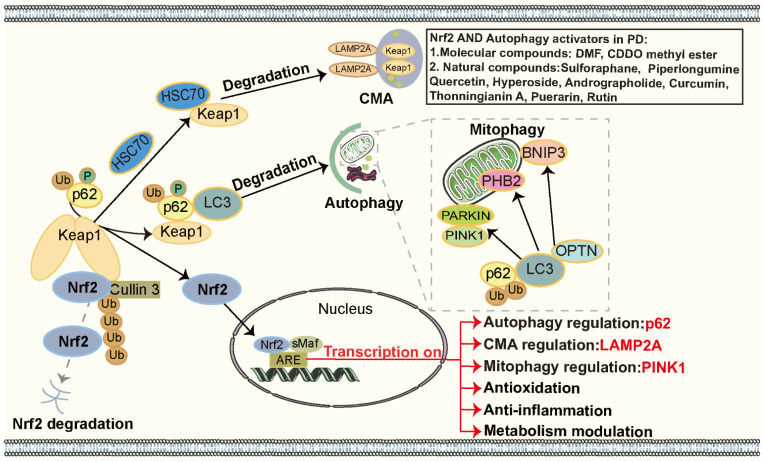
Mechanisms and therapeutic potential of the interplay between autophagy and the Nrf2 pathway in PD. In PD, autophagy facilitates the activation of the Nrf2 pathway by persistently degrading the LC3–p62–Keap1 complex. CMA and mitophagy also modulate Nrf2 activity in PD. For instance, HSC70, a molecular chaperone, binds to Keap1, directing it to LAMP2A for lysosomal degradation, thereby activating the Nrf2 pathway. Additionally, PHB2, a critical protein located in the inner mitochondrial membrane and integral to mitophagy, regulates Nrf2 expression and activity. In turn, the Nrf2 pathway reciprocally regulates autophagy by modulating the expression of key target genes in PD, including *p62*, *LAMP2A*, and *PINK1*. Numerous activators targeting both autophagy and the Nrf2 pathway synergistically alleviate dopaminergic neuronal injury, underscoring their potential as therapeutic strategies for PD.

In PD pathogenesis, either dysfunctional autophagy or elevated oxidative stress contributes to the aggregation of α-synuclein, resulting in the gradual loss of DAergic neurons [12,287]. We propose that autophagy dysfunction and oxidative stress may synergistically accelerate DAergic neuronal damage in PD pathology. Previous studies have revealed the intricate reciprocal regulation between autophagy and the Nrf2 antioxidant pathway in PD (Figure 5).

Numerous studies have clarified that autophagy induces the activation of the Nrf2 pathway in PD models. The ERK5/KLF4 signaling pathway mediates the autophagic removal of the p62–Keap1 complex, thus activating the Nrf2 antioxidant pathway and countering rotenone-induced neurotoxicity [36]. Andrographolide-induced autophagy mediates the mTORC1–p38–ERK pathway, leading to p62 phosphorylation and subsequent activation of Nrf2, which reduces α-synuclein aggregates, damaged mitochondria, and oxidative stress in MPP^+^-induced SH-SY5Y cells [38]. Similarly, thonningianin A, by activating Atg7-dependent autophagy, degrades Keap1 protein, promotes Nrf2 activation, and thus attenuates 6-OHDA-induced oxidative stress and ferroptosis in zebrafish and DAergic neurons [190]. In summary, the autophagic degradation of Keap1 activates the Nrf2 pathway, alleviating DAergic neuronal injury and delaying the progression of PD. Additionally, in SH-SY5Y cells, overexpression of p62 promotes Keap1 degradation, activates the Nrf2 pathway, increases HO-1 levels, neutralizes oxidative stress, and thus alleviates 6-OHDA-induced ferroptosis [190]. These studies underscore that targeting the p62–Keap1–Nrf2 axis could be a promising avenue for PD therapy.

Additionally, in 6-OHDA-induced PD models, impaired CMA, caused by LAMP2 knockdown or treatment with bafilomycin A1, hinders the degradation of Keap1, thereby leading to the inactivation of Nrf2. This suggests that CMA plays a crucial role in regulating the Nrf2 pathway in PD models [270]. Furthermore, in MPP^+^-induced SH-SY5Y cells, silencing PHB2 reduces Nrf2 levels, accelerates oxidative stress, and promotes cell death, indicating that mitophagy also regulates the Nrf2 pathway in PD models [272]. The above studies reveal that targeting mitophagy may also be a potential therapeutic option for PD.

Several studies have clarified that the activation of the Nrf2 pathway can trigger autophagy in PD. In wild-type mice overexpressing human α-synuclein, Nrf2 knockout results in dysfunctional autophagy, accompanied by increased phosphorylation and oligomerization of α-synuclein. Conversely, astrocyte-specific overexpression of Nrf2 mitigates oxidative stress, maintains normal neuronal autophagy, and alleviates α-synuclein aggregates, thereby protecting DAergic neurons from injury [288]. Although Nrf2 primarily exists in astrocyte and microglia, current studies reveal that Nrf2 can be activated in neurons under stress. For instance, in SH-SY5Y cells, mesencephalic astrocyte-derived neurotrophic factor activates the Nrf2 pathway, promoting the expression of lysosome-associated genes, which further triggers autophagy and reduces α-synuclein accumulation [40]. This study suggests that the neuronal Nrf2 pathway may also induce autophagy, thereby contributing to neuroprotection in PD. However, the mechanism by which the neuronal Nrf2 pathway regulates autophagy in PD pathology remains to be elucidated.

In addition, numerous Nrf2 activators can induce autophagy in PD. For instance, DMF promotes autophagosome formation, thereby combating rotenone-induced DAergic neurodegeneration [233]. Similarly, administration of the Nrf2 activator CDDO methyl ester attenuates neurodegeneration primarily through the activation of the Nrf2 pathway, which stimulates the antioxidant signaling pathway and enhances autophagy in rotenone-induced Drosophila PD and 6-OHDA-induced rat PD models [37]. However, the mechanisms by which the Nrf2 pathway, induced by CDDO methyl ester, triggers autophagy remain to be clarified. In conclusion, activation of the Nrf2 pathway, which subsequently triggers autophagy, may offer a promising therapeutic strategy for PD.

Furthermore, the Nrf2 pathway activates CMA, inhibits α-synuclein accumulation, and alleviates rotenone-induced DAergic neuron degeneration, suggesting that the Nrf2 pathway orchestrates CMA, thereby alleviating neurodegeneration in PD [40]. Notably, the Nrf2 pathway also induces mitophagy and contributes to DAergic neuroprotection in PD. Nrf2 triggers PINK1/PARKIN-induced mitophagy, reduces impaired mitochondria, and neutralizes oxidative stress in mice subjected to MPP^+^ iodide treatment [41]. Intriguingly, Nrf2 can also induce mitophagy in a PINK1/PARKIN-independent manner in PD. In a fruit fly model lacking PINK1/PARKIN, Nrf2 activation effectively triggers mitophagy and ameliorates neurodegeneration [289]. Additionally, administration of Nrf2 activator DMF has been shown to promote BNIP3-mediated mitophagy in PD models, although the precise molecular mechanisms underlying this process remain to be elucidated [41]. These findings underscore the multifaceted role of Nrf2 in modulating various subtypes of autophagy and highlight its potential as a therapeutic target for PD.

Natural compounds contribute to neuronal survival in PD models by activating both autophagy and the Nrf2 pathway (Figure 5). For instance, sulforaphane mitigates neurotoxicity by enhancing the Nrf2 antioxidant pathway, restoring autophagic function, and suppressing neuroinflammation and neuronal apoptosis [290]. Piperlongumine mitigates neurodegeneration in various PD models [291,292,293]. Mechanistically, it activates both autophagy and the Nrf2 pathway, thereby restoring motor coordination in rotenone-treated mice [292]. Additionally, piperlongumine modulates the NF-κB signaling pathway, reducing neuroinflammation in lipopolysaccharide-treated BV2 cells [293]. Quercetin activates the Nrf2 antioxidant pathway and PINK1/PARKIN-mediated mitophagy, decreasing α-synuclein levels, alleviating neuronal degeneration, and improving PD-related motor symptoms [294,295,296]. Hyperoside regulates both autophagy and the Nrf2 pathway, thus suppressing oxidative stress and reducing α-synuclein accumulation in PD models [297,298]. Andrographolide stimulates both autophagy and the Nrf2 pathway, facilitating the removal of α-synuclein and damaged mitochondria, while attenuating MPP^+^-induced neurotoxicity [38]. Furthermore, in lipopolysaccharide-MPP^+^ co-treated BV2 cells, andrographolide restores the compromised PARKIN-dependent autophagic flux, mitigating NLRP3 inflammasome activation and rescuing dopaminergic neuron loss [299]. Curcumin enhances mitochondrial function and antioxidant capacity by inducing p62–Keap1–Nrf2-mediated autophagy, thereby reversing motor deficits in rotenone-treated PD mice [27]. As noted previously, thonningianin A activates both autophagy and the Nrf2 pathway, alleviating 6-OHDA-induced neurotoxicity [190]. Puerarin activates the Nrf2 pathway by inhibiting GSK-3β/Fyn-mediated nuclear exclusion of Nrf2, thereby alleviating motor deficits in MPTP-induced mice [300]. Additionally, puerarin upregulates the expression of LAMP2A and HSC70, promoting CMA and mitigating oxidative stress in MPP^+^-treated SH-SY5Y cells [301]. Rutin induces PHB2-regulated mitophagy and Nrf2 activation, thereby ameliorating oxidative injury in PD models [272]. Collectively, these studies underscore the significance and therapeutic potential of the crosstalk between autophagy and the Nrf2 pathway in PD. However, further investigation into this interaction is needed.

## 6. Conclusions and Future Directions

Recently, the rising incidence of PD has intensified the societal demand for effective treatments. PD encompasses multiple pathological processes that interact to form a complex network driving its onset and progression. Consequently, targeting the crosstalk between these pathological processes may offer a promising therapeutic strategy. Both autophagy and the Nrf2 pathway are critical pathological processes in PD. Dysfunctional autophagy and an insufficient Nrf2 pathway may result in the initiation and progression of PD, respectively. Intriguingly, emerging studies reveal an intricate mutual regulation between autophagy and the Nrf2 pathway in PD. However, the precise mechanisms underlying this interplay remain to be clarified, particularly that by which the Nrf2 pathway regulates autophagy. Therefore, a deeper investigation into the interplay between autophagy and the Nrf2 pathway may offer novel comprehensive avenues for PD prevention and therapeutics.

In addition, numerous natural compounds and small-molecule activators have been found to induce both autophagy and the Nrf2 pathway, yet their specific mechanisms remain unclear. It is necessary to further screen and explore these compounds to clarify their mechanisms and evaluate their clinical application potential in PD therapeutics. Furthermore, considering that autophagy and the Nrf2 pathway are merely part of the broader PD pathological network, future investigations should aim to develop integrated multi-target therapeutic strategies to achieve more comprehensive neuroprotection.

## Data Availability

Not applicable.
